# pERK/pAkt phenotyping in circulating tumor cells as a biomarker for sorafenib efficacy in patients with advanced hepatocellular carcinoma

**DOI:** 10.18632/oncotarget.6104

**Published:** 2015-10-26

**Authors:** Jun Li, Lehua Shi, Xiaofeng Zhang, Bin Sun, Yefa Yang, Naijian Ge, Huiying Liu, Xia Yang, Lei Chen, Haihua Qian, Mengchao Wu, Zhengfeng Yin

**Affiliations:** ^1^ Eastern Hepatobiliary Surgery Hospital, Second Military Medical University, Shanghai, China

**Keywords:** ERK/Akt, circulating tumor cells, hepatocellular carcinoma, sorafenib

## Abstract

Sorafenib is a multikinase inhibitor approved for the treatment of advanced hepatocellular carcinoma (HCC). However, therapeutic response to sorafenib was not equal among HCC patients. Here we present a novel system to provide quantitative information concerning sorafenib-related targets by simultaneous detection of phosphorylated ERK (pERK) and pAkt expressions in circulating tumor cells (CTCs) isolated from HCC patients. Our results showed that 90.0% of patients had a molecular classification of tissues concordant with that of CTCs. CTC counts showed a shaper decline in patients with pERK^+^/pAkt^−^ CTCs after two weeks of sorafenib treatment (*P* < 0.01). Disease control rates were significantly different between patients with pERK^+^/pAkt^−^ CTCs (11/15; 73.3%) and those without (13/44; 29.5%) (*P* < 0.05). Univariate and multivariate analysis indicated pERK^+^/pAkt− CTCs as an independent predictive factor of progression-free survival (PFS) (hazard ratio = 9.389; *P* < 0.01). PFS correlated with the proportion of pERK^+^/pAkt^−^ CTCs (*r* = 0.968, *P* < 0.01), and was higher in patients with ≥ 40% pERK^+^/pAkt− CTCs compared to those with < 40% (8.4 *vs*. 1.3 mo; *P* < 0.05). In a validation set of twenty HCC patients, CTCs from patients with ≥ 40% pERK^+^/pAkt^−^ CTCs had significantly higher inhibition rates of spheroid formation compared to those with < 40% (61.2 *vs*. 19.8%; *P* < 0.01). Our findings demonstrated that CTCs can be used in place of tumor tissue for characterization of pERK/pAkt expression. pERK^+^/pAkt^−^ CTCs are most sensitive to sorafenib and an independent predictive factor of PFS in HCC patients treated with sorafenib.

## INTRODUCTION

Hepatocellular carcinoma (HCC) is one of the most common malignancies worldwide, for which the prognosis remains poor, particularly for those with advanced disease [1]. The recent approval of sorafenib as the first effective oral drug for HCC marks a significant milestone in the treatment of this disease [2]. Sorafenib is a multitargeted, small molecule tyrosine kinase inhibitor with multiple antitumor effects, including antiangiogenic, antiproliferative, and pro-apoptotic effects via inhibition of vascular endothelial growth factor receptor (VEGFR)-1, -2 and -3, platelet-derived growth factor receptor (PDGFR) β, Raf-1, B-Raf, and C-Raf [3]. Large randomized phase III studies indicate that sorafenib treatment improves the survival of patients with advanced HCC [4–6]. However, the response rate of sorafenib is quite low, and not all patients respond equally well to sorafenib treatment [4, 5], for reasons that are not entirely clear.

A variety of studies have shown that the inactivation of Ras/Raf/extracellular signal-regulated kinase (ERK) pathway and the activation of the phosphoinositide 3-kinase (PI3K)/protein kinase B (Akt)/mammalian target of rapamycin (mTOR) pathway in tumors play a critical role in the resistance to sorafenib [7–14]. Thus, these pathways may provide biomarkers to assess sorafenib-resistance before and throughout the course of treatment.

Currently, tumor tissues obtained by an excisional or needle biopsy are used for detection of drug targets. However, needle aspiration often fails to locate measurable objectives or obtain sufficient tumor samples, and only a small proportion of patients are eligible for surgical excision at diagnosis. Furthermore, invasive sampling is potentially harmful and expensive, and cannot be performed repeatedly. The collection of circulating tumor cells (CTCs) provides a viable alternative. CTCs are cancer cells shed from either the primary tumor or its metastases that circulate in the peripheral blood, which thus are available noninvasively and can be obtained repeatedly for a readily accessible real-time “liquid biopsy” of tumors [15]. Molecular characterization of CTCs has been used in the development of personalized targeted therapies in breast, lung, prostate, and colorectal cancer [16–21]. It is worth noting that there have been a considerable number of studies which investigated phospho-proteins expressed in CTCs and revealed their biological significance, such as pAkt [22], pSRC [23], pEGFR [24, 25], pFAK and pPI3K [26]. Also, pAkt and pERK have been widely studied in HCC tissues over a period of years [7–14, 27]. Thus, we utilized CTCs to evaluate the activation (phosphorylation) of ERK and Akt with sorafenib for treatment of HCC, and to determine if pERK/pAkt phenotyping of CTCs can be used as a viable diagnostic biomarker for sorafenib efficacy.

## RESULTS

### Classification of HCC patients via pERK/pAkt expression patterns in CTCs

**Figure 1:**
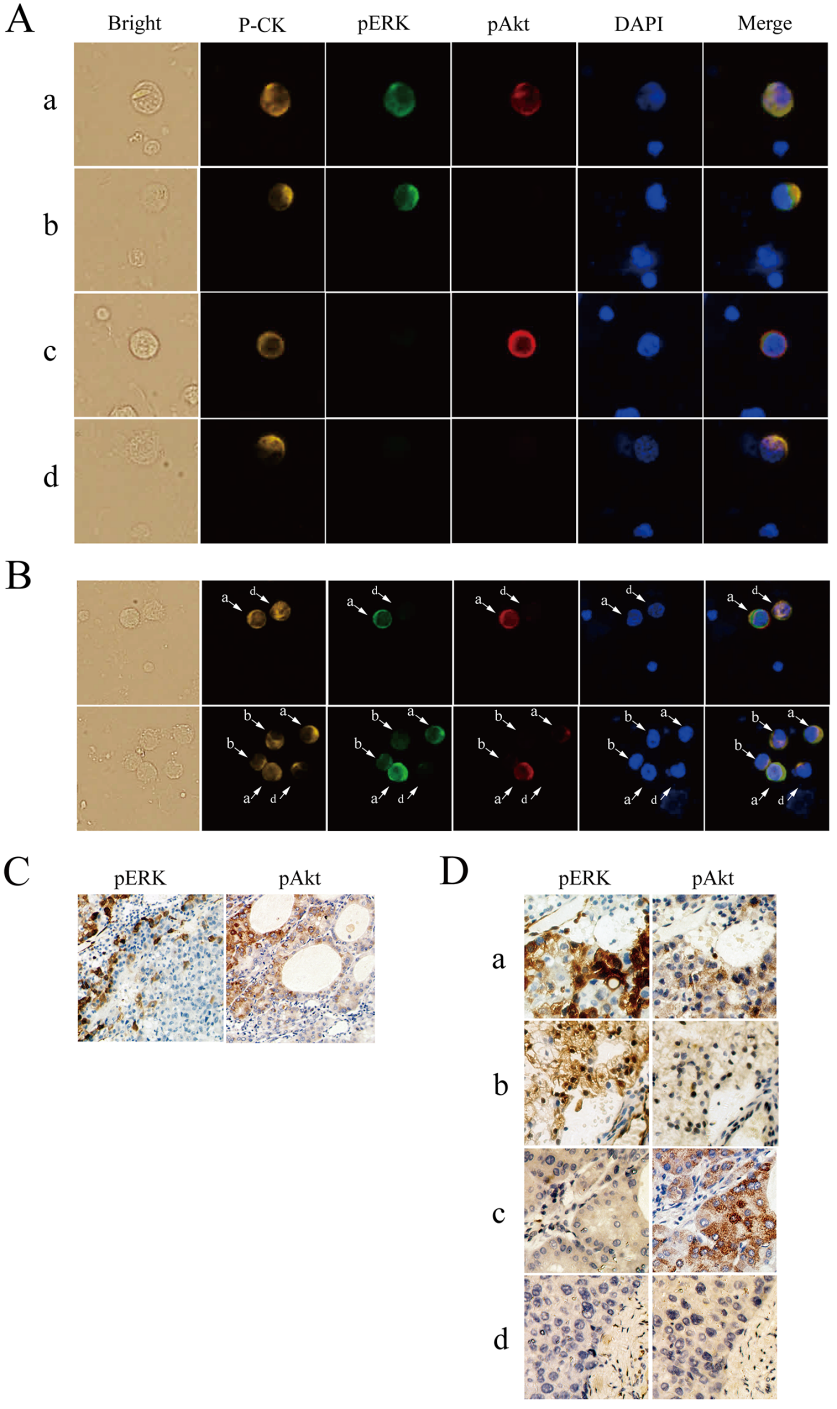
Detection of phosphorylated extracellular signal-regulated kinase (pERK) and protein kinase B (pAkt) in hepatocellular carcinoma **A.** Circulating tumor cells (CTCs) stained for pan-cytokeratin (P-CK) (yellow), pERK (green), pAkt (red), and costained with 4′,6-diamidino-2-phenylindole (DAPI) (blue) (400× magnification). **B.** Coexistence of CTCs with various patterns of pERK/pAkt in the same field of view detected by multicolor immunofluorescence staining (200×). **C.** Immunohistochemical staining for pERK and pAkt in an individual cancer tissue (100×). **D.** Immunohistochemical staining for pERK and pAkt in serial sections of hepatocellular carcinoma tissues (200×); a, pERK^+^/pAkt^+^; b, pERK^+^/pAkt^−^; c, pERK^−^/pAkt^+^; d, pERK^−^/pAkt^−^.

CTC counts ranged from 0–137 per 5 mL of blood, and were detected in 101/109 (92.7%) patients with advanced HCC (52 ± 23 CTCs/5 mL). Immunofluorescence staining revealed specific and heterogeneous cytoplasmic expression of pERK and pAkt in CTCs (Fig. 1A); 25/101 (24.8%) patients had pERK^+^ CTCs, and 81/101 (80.2%) had pAkt^+^ CTCs. Generally, CTCs with various patterns of pERK/pAkt expression were found within blood samples from the same patient (Fig. 1B), some of which even contained all four possible phenotypes (pERK^+^/pAkt^+^, pERK^+^/pAkt^−^, pERK^−^/pAkt^+^, and pERK^−^/pAkt^−^). Patients were defined as pERK or pAkt positive based on the presence of pERK^+^ or pAkt^+^ CTCs, and then classified into four subsets according to the combination of pERK and pAkt positivity (Supplementary Table S1).

### Concordance of pERK/pAkt expression in CTCs and tumor tissues

pERK and pAkt were detected in tumor tissue specimens, but the expression was not uniform throughout (Fig. 1C). According to positivity-defining criteria as previously described [9], 7/32 (21.9%) tumors were classified as pERK^+^ and 27/32 (84.4%) were pAkt^+^, which were similar to the 24.8% and 80.2%, respectively, determined from CTC analyses. In addition, all four phenotype patterns of pERK/pAkt expression were observed in tissue specimens (Fig. 1D). The molecular classification based on analysis of both tumor tissues and CTCs in 32 patients is summarized in Table 1. CTCs were detected in 30/32 patients from whom tissue specimens were obtained, and molecular classification of CTC and tissue was concordant in 27/30 (90.0%) patients.

**Table 1:** pERK/pAkt phenotyping of tumor tissues and circulating tumor cells (CTCs) in patients with hepatocellular carcinoma, *n* = 32

Tumor tissues	CTCs					
	pERK^+^/pAkt^+^	pERK^+^/pAkt^−^	pERK^−^/pAkt^+^	pERK^−^/pAkt^−^	Not detected	Total
pERK^+^/pAkt^+^	4	0	0	0	0	4
pERK^+^/pAkt^−^	1	2	0	0	0	3
pERK^−^/pAkt^+^	1	0	20	0	2	23
pERK^−^/pAkt^−^	0	1	0	1	0	2
Total	6	3	20	1	2	32

Note: Total concordance is 90% (27/30).

### Distribution and percentage of pERK/pAkt phenotypes in CTCs

The molecular classification of all 101 patients with HCC that were categorized according to pERK or pAkt positivity in CTCs is presented in Table 2. The distribution and percentage of CTC phenotypes varied among individuals, even individuals within the same subset, and multiple phenotypes could be found within an individual.

**Table 2:** Molecular classification of hepatocellular carcinoma based on pERK/pAkt phenotypes of circulating tumor cells (CTCs), *n* = 101

Patient classification	CTC phenotypes	Patients (*n*)	Total (*n*)
PP	PP	1	16
	PP, PN	2	
	PP, NP	0	
	PP, NN	2	
	PN, NP	2	
	PP, PN, NP	2	
	PP, PN, NN	1	
	PP, NP, NN	1	
	PN, NP, NN	3	
	PP, PN, NP, NN	2	
PN	PN	4	9
	PN, NN	5	
NP	NP	16	65
	NP, NN	49	
NN	NN	11	11

Abbreviations: NN, pERK^−^/pAkt^−^; NP, pERK^−^/pAkt^+^; PN, pERK^+^/pAkt^−^; PP, pERK^+^/pAkt^+^.

### Sorafenib response correlates with pERK^+^/pAkt− CTCs in HCC Patients

**Figure 2:**
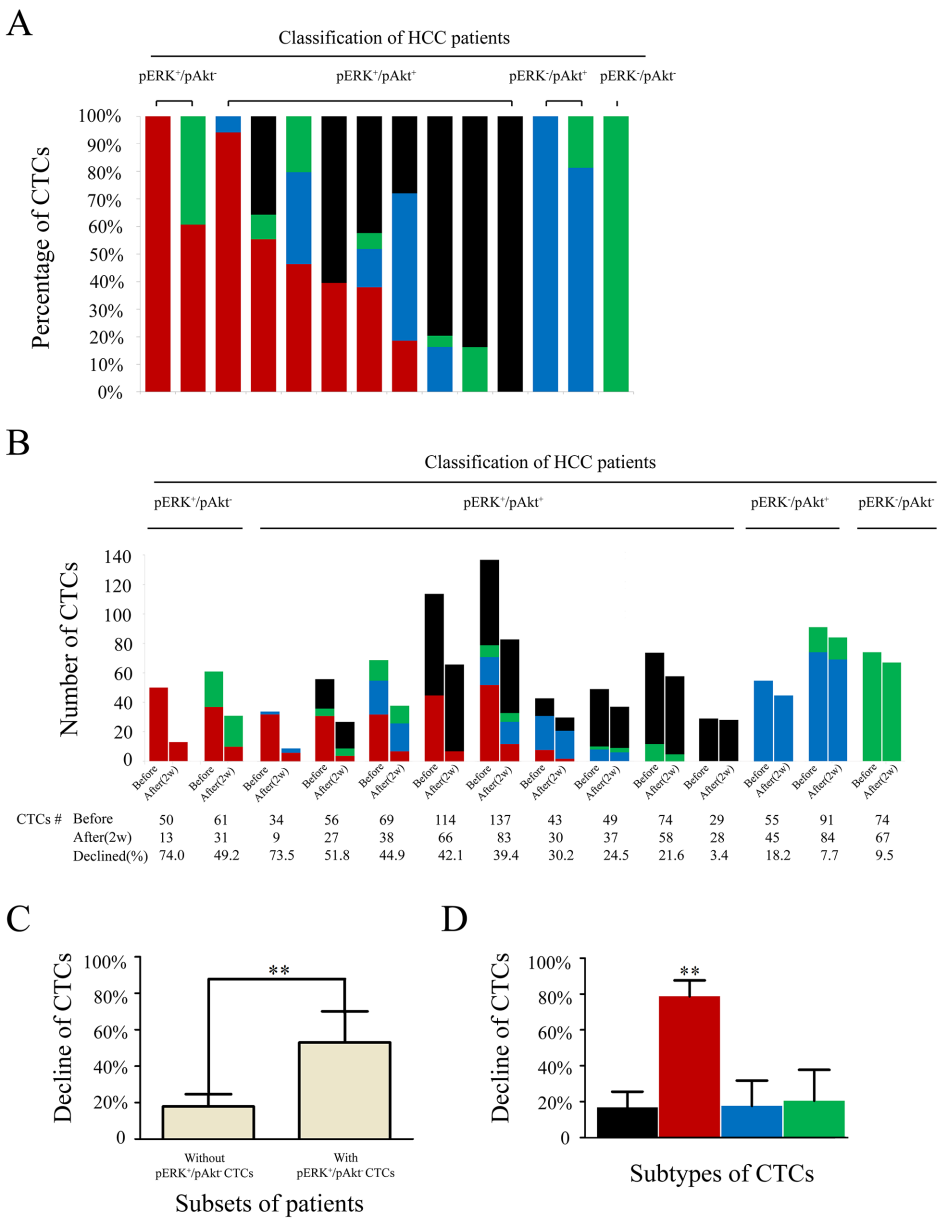
Numbers and percentages of circulating tumor cell (CTC) subtypes/total in hepatocellular carcinoma patients receiving sorafenib treatment **A.** Percentages of CTC subtypes before treatment in 14 randomly selected patients from each subgroup. **B.** CTC counts in 14 patients before and after receiving sorafenib treatment. **C.** Changes in total CTCs after sorafenib treatment in patients with (*n* = 15) compared to those without (*n* = 44) pERK^+^/pAkt^−^ CTCs. **D.** Changes in CTC subtypes in 59 patients receiving sorafenib treatment; black, pERK^+^/pAkt^+^; red, pERK^+^/pAkt^−^; blue, pERK^−^/pAkt^+^; green, pERK^−^/pAkt^−^ CTCs.

A total of 64 patients received sorafenib monotherapy, which was interrupted in five due to intolerable adverse reactions, including hand-foot syndrome (*n* = 2), diarrhea (*n* = 2), and vomiting (*n* = 1). Therefore, 59 patients were included in evaluation of sorafenib efficacy. All patients suffered varying rates of decline in CTC counts after two weeks of treatment, from 56 ± 20 to 41 ± 18 CTCs/5 mL of blood. Pretreatment percentages of different CTC subtypes in at least one patient randomly selected from each subgroup with varied CTCs subtypes are shown in Fig. 2A. As shown in Fig. 2B, pERK^+^/pAkt^−^ (*n* = 2) and pERK^+^/pAkt^+^ (*n* = 9) classified patients suffered a severe decrease in CTC counts; and particularly patients with pERK^+^/pAkt^−^ CTCs (*n* = 8). CTC counts pre- and post-treatment with sorafenib in each subset of patients were listed in Supplementary Table S2. Further analysis indicated that the decline in CTC counts in patients with pERK^+^/pAkt^−^ CTCs (*n* = 15) was 53.0 ± 17.1% (range: 30.2–83.3%), which was significantly higher than in those without (*n* = 44), for whom CTC counts declined 17.9 ± 6.7% (range: 3.4–32.6%) (*P* < 0.01) (Fig. 2C).

In all 59 patients, the decline in pERK^+^/pAkt^−^ CTCs (78.7 ± 8.9%) was significantly greater than in pERK^+^/pAkt^+^ (16.9 ± 8.7%), pERK^−^/pAkt^+^ (17.6 ± 14.2%), and pERK^−^/pAkt^−^ (20.5 ± 17.3%) CTCs (*P* < 0.01) (Fig. 2D).

**Figure 3:**
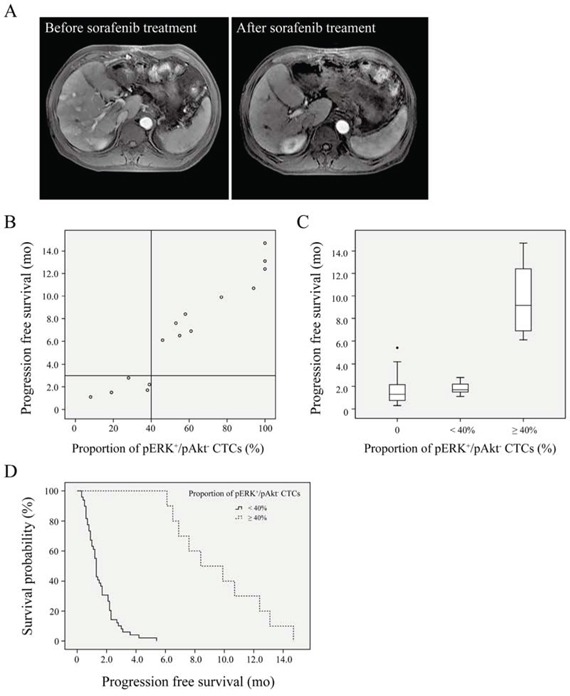
Survival curves for hepatocellular carcinoma patients **A.** Dynamic contrast-enhanced magnetic resonance imaging. Several tumor nodules with gadolinium-DTPA enhancement located in both the left and right lobes were shrunken or had completely disappeared after sorafenib treatment in a patient who only exhibited pERK^+^/pAkt^−^ CTCs. **B.** Progression-free survival after sorafenib treatment in patients (*n* = 15) according to (pERK^+^/pAkt^−^)/total circulating tumor cells (CTCs). **C.** Progression-free survival in patients with ≥ 40% (*n* = 10) or < 40% (*n* = 5) of CTCs identified as pERK^+^/pAkt^−^, and those without pERK^+^/pAkt^−^ CTCs (*n* = 44). **D.** Survival curves of patients with ≥ 40% (*n* = 10) or < 40% (*n* = 49) of CTCs identified as pERK^+^/pAkt^−^.

mRECIST evaluation revealed no significant correlations of tumor response with clinical characteristics (Table 3). CR, PR, SD, and PD was observed in 1/59 (1.7%), 11/59 (18.6%), 12/59 (20.3%) and 35/59 (59.3%) patients, respectively, resulting in a total DCR of 40.7%. The decline in CTC counts in patients with DCR (*n* = 24) was 39.5 ± 21.4% (range: 17.5–83.3%), which was significantly higher (*P* < 0.01) than 18.2 ± 9.1% (range: 3.4–42.1%) in patients with PD (*n* = 35). In the patients with pERK^+^/pAkt^−^ CTCs, the DCR was 73.3%, while this number was only 29.5% in those without (*P* = 0.003). Further analysis showed no significant positive correlation of tumor response with CTC phenotypes other than pERK^+^/pAkt− CTCs (Table 3). An example of tumor response to sorafenib is shown in a 59-year-old man with recurrent HCC, who only possessed pERK^+^/pAkt^−^ CTCs and had several tumor nodules with gadolinium-DTPA enhancement located in both the left and right lobes; after treatment with sorafenib for 8 mo, these tumors were shrunken or had completely disappeared as evidenced by dynamic contrast-enhanced MRI (Fig. 3A).

**Table 3:** Disease control rates (DCR) and clinical characteristics of 59 patients with hepatocellular carcinoma receiving sorafenib monotherapy

Variable	Patients (*n*)					DCR(%)	*P*
	Total	CR	PR	SD	PD		
Sex							
Male	44	1	9	10	24	45.5	0.201
Female	15	0	2	2	11	26.7	
Age, y							
> 50	40	1	8	10	21	47.5	0.122
≤ 50	19	0	3	2	14	26.3	
HBV							
Positive	50	1	10	10	29	42.0	0.626
Negative	9	0	1	2	6	33.3	
Maximum tumor size, cm							
> 3	34	1	7	6	20	41.2	0.928
≤ 3	25	0	4	6	15	40.0	
AFP level, ng/mL							
< 400	37	1	9	6	21	43.2	0.603
≥ 400	22	0	2	6	14	36.4	
ECOG PS							
0	14	0	3	5	6	57.1	0.151
1 or 2	45	1	8	7	29	35.6	
Child–Pugh class							
A	42	1	8	10	23	45.2	0.262
B	17	0	3	2	12	29.4	
Portal vein thrombus							
Positive	48	1	8	9	30	37.5	0.299
Negative	11	0	3	3	5	54.5	
TNM staging							
III	46	1	9	11	25	45.7	0.143
IV	13	0	2	1	10	23.1	
Number of CTCs							
≤ 53^a^	31	1	8	7	15	51.6	0.072
> 53	28	0	3	5	20	28.6	
pERK^+^/pAkt^+^ CTCs							
Present	8	0	1	2	5	37.5	0.844
Absent	51	1	10	10	30	41.2	
pERK^+^/pAkt^−^ CTCs							
Present	15	1	7	3	4	73.3	0.003
Absent	44	0	4	9	31	29.5	
pERK^−^/pAkt^+^ CTCs							
Present	40	0	4	9	27	32.5	0.064
Absent	19	1	7	3	8	57.9	
pERK^−^/pAkt^−^ CTCs							
Present	44	0	7	11	26	40.9	0.951
Absent	15	1	4	1	9	40.0	

Abbreviations: AFP, alpha-fetoprotein; CR, complete response; CTC, circulating tumor cell; HBV, hepatitis B virus; PD, progressive disease; PR, partial response; PS, performance status; SD, stable disease; TNM, tumor-node-metastasis stage.

Note:

a 53 was the median number of CTCs detected in 59 HCC patients.

### Proportion of pERK^+^/pAkt^−^ CTCs as a potential predictive factor of HCC patients treated with sorafenib

Univariate analysis of predictive factors for PFS indicated that pERK^+^/pAkt^−^ CTCs but not other types of CTCs was significantly associated with PFS (Table 4). The multivariate analysis using both significant and near-significant variables (up to *P* = 0.1 in the univariate analysis) showed that pERK^+^/pAkt^−^ CTCs remained an independent factor associated with a good prognosis (hazard ratio = 9.389, *P* < 0.01). CTC phenotypes and PFS for 15 patients with pERK^+^/pAkt^−^ CTCs are listed in Supplementary Table S3. Spearman rank correlation analysis showed that PFS was correlated with the proportion of CTCs identified as pERK^+^/pAkt^−^ (r = 0.968; *P* < 0.01), but not with the number of pERK^+^/pAkt^−^ CTCs (*r* = 0.491). Moreover, patients with *a* ≥ 40% proportion of pERK^+^/pAkt^−^ CTCs (*n* = 10) had a longer PFS than those with < 40% (*n* = 49) (Fig. 3B, 3C), which was confirmed by Kaplan-Meier analysis and log-rank test (median PFS: 8.4 [95% CI: 4.8–12.0] *vs.* 1.3 [95% CI: 1.2–1.4] mo) (Fig. 3D).

**Table 4:** Univariate and multivariate analysis of predictive factors for progression-free survival

Factors	Progression-free survival			
	Univariate	Multivariate		
	*P* value	HR	95% CI	*P* value
Sex: male *vs*. female	0.648			NA
Age: > 50 *vs*. ≤ 50 years	0.277			NA
HBV: positive *vs*. negative	0.378			NA
Maximum tumor size: > 3 *vs*. ≤ 3 cm	0.298			NA
AFP level: < 400 *vs*. ≥ 400 ng/mL	0.449			NA
ECOG PS: 0 *vs*. 1–2	0.586			NA
Child-Pugh class: A *vs*. B	0.065	1.024	0.551–1.902	0.941
Portal vein thrombus:positive *vs*. negative	0.828			NA
TNM staging: III *vs*. IV	0.066	0.609	0.296–1.253	0.178
Number of CTCs: > 53 *vs*. ≤ 53^a^	0.275			NA
pERK^+^/pAkt^+^ CTCs: present *vs*. absent	0.647			NA
pERK^+^/pAkt^−^ CTCs: present *vs*. absent	< 0.001	9.389	3.242–27.192	< 0.001
pERK^−^/pAkt^+^ CTCs: present *vs*. absent	0.061	1.129	0.523–2.437	0.757
pERK^−^/pAkt^−^ CTCs: present *vs*. absent	0.132			NA

Abbreviations: AFP, alpha-fetoprotein; CTC, circulating tumor cell; HBV, hepatitis B virus; TNM, tumor-node-metastasis stage; HR, hazard ratio; CI, confidence interval; NA, not adopted.

Note:

a 53 was the median number of CTCs detected in 59 HCC patients.

### pERK^+^/pAkt^−^ CTCs Are most sensitive to sorafenib *in vitro*

**Figure 4:**
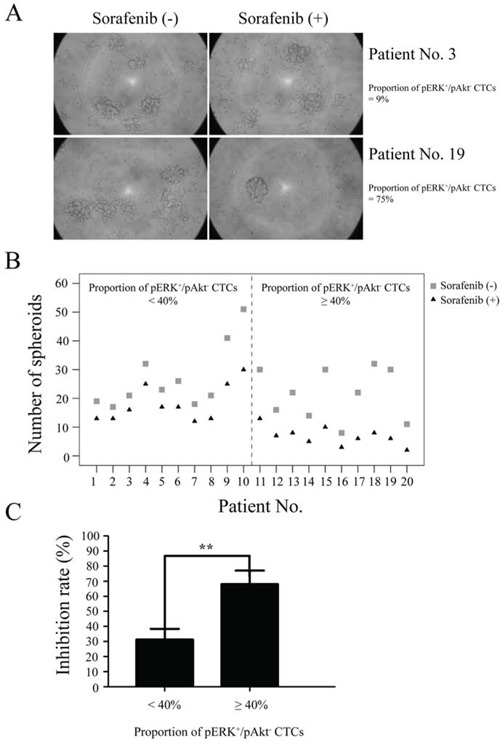
Sensitivity of circulating tumor cells (CTCs) to sorafenib CTCs isolated from hepatocellular carcinoma patients (*n* = 20) were tested by spheroid formation assay. **A.** CTCs from two patients formed spheroids at day 7 in culture with or without sorafenib. **B.** Formation of spheroids of CTCs treated with or without sorafenib. **C.** Spheroid formation inhibition rates from patients with ≥ 40% or < 40% of CTCs identified as pERK^+^/pAkt^−^.

An independent validation set of 20 HCC patients with similar clinical features was used to evaluate CTC sensitivity to sorafenib, including ten with < 40% pERK^+^/pAkt^−^ CTCs and ten with ≥ 40%. Fig. 4A shows two examples of sorafenib sensitivity tests where CTCs from two patients formed spheroids at day 7 in 3D culture with or without sorafenib. All 20 patients showed a decline in the number of spheroids formed when sorafenib was added to the culture medium. However, samples comprised of ≥ 40% pERK^+^/pAkt^−^ CTCs showed a larger decline (Fig. 4B) and had a significantly higher inhibition rate than those with < 40% (68.0 *vs*. 31.3%; *P* < 0.01) (Fig. 4C).

## DISCUSSION

Several studies have attempted to utilize serum biomarkers to predict response to sorafenib. Ten plasma biomarkers were prospectively evaluated in the SHARP trial, but failed to predict response to sorafenib [28]. Miyahara *et al* [29] analyzed nine serum cytokines in 30 HCC patients treated with sorafenib and observed that high levels of serum cytokines at baseline correlated with poor treatment response. However, the treatment response deteriorated as the number of cytokines at a high level increased. Disease progression was only seen in 25% patients with 0–2 high biomarkers and 33.3% with 3–5. Two recent independent studies by Gyöngyösi *et al* [30] and Vaira *et al* [31] suggested that microRNAs in HCC tissues could be used to assess expected survival of patients treated with sorafenib. The prognostic impact of microRNAs in response to sorafenib may be non-specific, as bioinformatics analysis did not identify any interaction between the microRNA gene targets and sorafenib activity or metabolism [31]. Other molecular markers such as Mcl-1 [32] and CD44 [33] may also have similar impact on response to sorafenib.

Although inhibition of VEGFR- or PDGFR-regulated intracellular kinase pathways in endothelial cells or pericytes is only one of multiple mechanisms of sorafenib action, expression levels of these growth factors or relevant receptors in HCC tissues have been evaluated to predict the clinical outcome of patients receiving sorafenib [34–39]. Moreover, a few studies have evaluated pERK expression before treatment in tissue biopsies or cell lines and found a positive correlation with a more favorable response to sorafenib [7, 8, 27].

Our results show concordance between CTCs and tumor tissues regarding pERK/pAkt phenotype status, with expression in CTCs similar to those reported in liver cancer tissues [7–14]. These data led us to believe that it is necessary to conduct target detection before the administration of sorafenib, and CTCs represent an effective, noninvasive method to collect samples. pERK/pAkt phenotyping in individual CTCs eliminates the interference from a large number of various components in the tissue, and more accurately reflects the true nature of a tumor. Analysis of CTCs provides comprehensive details concerning the specific phenotypes expressed and their proportions, which is unachievable when tumor tissues are used for detection.

We further investigated whether such quantitative information could be used to predict response to sorafenib in patients with HCC to personalize this therapeutic approach. All patients showed a decrease in CTC counts after two weeks of sorafenib monotherapy. The greatest decrease occurred in patients with a high proportion of pERK^+^/pAkt^−^ CTCs, suggesting that this phenotype is most sensitive to sorafenib, and likely the reason for the clinical efficacy of sorafenib treatment. The slight decrease in other CTC phenotypes probably resulted from a tumor burden reduction by antiangiogenic or other mechanisms of sorafenib.

As HCC is a hypervascular cancer, it was theorized that the efficacy of sorafenib is primarily attributable to its inhibition of tumor angiogenesis by targeting VEGFR and PDGFR, and partly to the inhibition of Raf [40]. Indeed, several angiogenesis inhibitors (sunitinib, linifanib and brivanib) targeting similar pathways have been compared with sorafenib in Phase III trials, however these drugs failed to demonstrate superior efficacy in patients with HCC compared with the standard treatment [41–44]. Notably, a study in an orthotopic HCC mouse model demonstrated that sorafenib had a pro-metastasis effect in HCC through downregulation of tumor suppressor *HTATIP2*, which is associated with inhibition of angiogenesis [45]. Therefore, we speculate that targeting HCC cells by inhibiting the Ras/Raf/ERK signaling pathway, rather than targeting endothelial cells by inhibiting angiogenesis, is probably the primary contribution to the anti-tumor efficacy of sorafenib. Moreover, the resistance to sorafenib determined by specific molecular features of HCC cells in most patients will decrease its overall clinical efficacy, as shown by our study and others [4, 5, 7–13].

Importantly, our study demonstrates that therapeutic response to sorafenib, as well as PFS, only correlate with the proportion of pERK^+^/pAkt− CTCs, particularly when the proportion is ≥ 40%. This result was somewhat inconsistent with the present knowledge of CTCs, which indicates that patients with a higher CTC count have a higher tumor burden, higher tumor stage, and demonstrate poorer therapeutic efficacy. However, our data are supported by the observation that sorafenib significantly inhibited spheroid formation of pERK^+^/pAkt^−^ CTCs. Previous reports have shown that overall survival is paradoxically prolonged for patients who receive a half-dose of sorafenib for 70% of the treatment period compared to those who maintained full dosing or had a dose reduced for < 70% of the treatment period [4, 46]. It is possible that the cumulative dose was increased in patients who underwent a dose reduction due to fewer side effects, better tolerance, and prolonged treatment exposure. Indeed, in the study by Iavarone *et al* [46] the dose of sorafenib was only reduced as required by intolerance or side effects, which typically denote better susceptibility to the drug. Thus, the efficacy of a drug could be higher in a patient that experiences side effects, regardless of dose reduction. This implies that targeted drugs may not follow the therapeutic paradigm of cytotoxic anticancer drugs, for which clinical activity correlates with dosing and achievement of clinically effective blood levels [47]. This may also explain why sorafenib response did not correlate with clinical characteristics or total CTC count in our study. The response was mainly dependent on inherent sensitivity or resistance to sorafenib. On the other hand, a higher number but a lower percentage of pERK^+^/pAkt^−^ CTCs in a single patient indicates that more tumor cells are resistant to sorafenib, thus generating poor clinical effectiveness, whereas a patient with a low number but higher percentage of pERK^+^/pAkt− CTCs may achieve a better outcome after sorafenib treatment.

In summary, this study presents a unique platform to provide quantitative information concerning sorafenib-related targets in CTCs, define the molecular subtypes of HCC to identify patients particularly susceptible to sorafenib, predict drug response and efficacy, and select patients most likely to benefit. Thus, CTC pERK/pAkt phenotyping will ultimately increase the success of sorafenib treatment, while preventing unnecessary treatments, serious side effects, and high costs. However, further large-scale clinical prospective investigations are needed to confirm and verify the clinical significance indicated by our study.

## MATERIALS AND METHODS

### Patients and sample collection

The study enrolled 109 patients with advanced HCC who were candidates for sorafenib treatment, including newly diagnosed patients (*n* = 50) and patients with recurrent HCC after surgical resection, transcatheter arterial chemoembolization or radiofrequency ablation (*n* = 59). Clinical characteristics of all patients are presented in Supplementary Table S4. Serial tissue sections were obtained from 32 patients who underwent surgical resection. Peripheral blood (5 mL) was collected in polyethylene tubes containing EDTA (Greiner Bio-One GmbH, Frickenhausen, Germany) after discarding the first 5 mL of blood. The study was approved by the Biomedical Ethics Committee of Eastern Hepatobiliary Surgery Hospital (Shanghai, China) and informed written consent was obtained from all patients.

### Immunohistochemistry

Serial tissue sections were incubated with either mouse anti-human pAkt1/2/3 (1:100 dilution, Thr308/Thr309/Thr305; Santa Cruz Biotechnology, Dallas, TX, USA) or rabbit anti-human pERK1/2 (1:200 dilution, Thr202/Tyr204; Cell Signaling Technology, Danvers, MA, USA) monoclonal antibody at 4°C overnight, and then incubated with a horseradish peroxidase-conjugated secondary antibody (Maixin-Bio, Fuzhou, China) at room temperature for 45 min. The immunoreactivity was visualized using diaminobenzidine substrate (Maixin-Bio). The positivity was determined independently by three liver pathologists considering both intensity and subcellular localization as reported (9).

### CTC enrichment and multicolor immunofluorescence staining

CTC enrichment of whole blood samples was conducted according to the method previously described [48]. Briefly, following density gradient centrifugation, CTCs were enriched by extracting CD45-expressing leukocytes with magnetically labeled anti-CD45 monoclonal antibody according to the instructions. The remaining cells were cytocentrifuged on polylysine-coated slides. Slides prepared from blood samples were co-incubated with pAkt1/2/3 and pERK1/2 antibody at 37°C for 1 h, followed by Alexa Fluor 647-conjugated goat anti-mouse and FITC-conjugated goat anti-rabbit IgG antibody (1:500 dilution, Beyotime, Shanghai, China). Slides were subsequently stained with Alexa Fluor 555-conjugated pan-cytokeratin (P-CK) mouse monoclonal antibody (1:50 dilution, Cell Signaling Technology) and costained with 4′,6-diamidino-2-phenylindole (DAPI). As controls, slides prepared with PLC/PRF/5, Hep3B, HepG2, Huh7, MHCC-97H and -97L HCC cell lines were stained with primary antibodies or isotype control antibodies according to the method previously described [48].

### Identification and enumeration of CTCs with different phenotypes

Stained slides were viewed through a fluorescence microscope (IX71; Olympus, Tokyo, Japan), and images were captured from positively stained CTCs and control slides with the same gain and exposure time. P-CK and DAPI-stained cells that met morphologic features of malignant cells (large cellular size, high nuclear to cytoplasmic ratio, and visible nucleoli) were scored as CTCs. pAkt1/2/3 and pERK1/2 expression was examined in P-CK-positive CTCs. Cell counts are expressed as the number of cells per 5 mL of blood.

### Sorafenib-sensitivity testing

The sensitivity of CTCs to sorafenib was assessed using a three-dimensional (3D) cancer model for drug evaluation that mimics *in vivo* responses to drugs [49]. Briefly, CTCs isolated from 5 mL peripheral blood were resuspended in 150 μL DMEM containing sorafenib pre-dissolved in DMSO. Matrigel (Becton, Dickinson, and Company, Franklin Lakes, NJ, USA) was thawed and mixed equally with the CTC-containing DMEM. The prepared mixture was then incubated in a 24-well plate for 30 min at 37°C. Then, 500 μL of DMEM with sorafenib was added on top of the gel to give a final concentration of 10 μM sorafenib in 0.4% DMSO. The final concentration was estimated by sorafenib sensitivity tests for HCC cell lines to be the optimal concentration. Spheroid formation was observed every day and counted on day 7. A spheroid was defined as 3D cell structure > 100 μm in diameter.

### Sorafenib treatment

The final clinical decision for sorafenib treatment, and as a monotherapy or combined, was made by physicians blind to the CTC detection results. Patient inclusion criteria included: proven diagnosis either by histological or characteristic radiologic or serologic findings; ineligibility for or progression after surgical resection; Child-Pugh liver function class A or B; Eastern Cooperative Oncology Group (ECOG) performance status 0–2; adequate hematological functions (white blood cells > 4 × 10^9^/L or absolute neutrophil count > 1.5 × 10^9^/L, platelets > 100 × 10^9^/L, hemoglobin > 10 g/dL); and preserved organ functions (serum creatinine level < 1.5 mg/dL, serum alanine and aspartate aminotransferase levels < 5 times the upper limit of the normal range). An initial dose of 400 mg sorafenib b.i.d. (Nexavar; Bayer HealthCare Pharmaceuticals, Leverkusen, Germany) was administered, and treatment was interrupted or dosage was reduced for drug-related adverse effects. Sorafenib administration was continued until intolerable toxicity or disease progression occurred.

### Outcomes and assessments

A total of 59 patients were included for efficacy assessments. An additional 5 mL sample of peripheral blood was collected two weeks after sorafenib administration and processed as described above. Tumor sizes were measured at baseline and every 4–8 weeks during treatment by dynamic contrast-enhanced CT or MRI. Response to sorafenib in patients was evaluated using the modified Response Evaluation Criteria in Solid Tumors (mRECIST) and classified as complete response (CR), partial response (PR), stable disease (SD), or progressive disease (PD). Disease control rate (DCR) was defined as the percentage of cases showing CR, PR, or SD. Progression-free survival (PFS) was calculated as the time from the first cycle of sorafenib to radiologic or serologic progression or death from any cause. Survival was censored if a change in therapy occurred.

### Statistical analysis

The statistical analyses were performed using SPSS software (version 17.0; SPSS Inc., Chicago, IL, USA) and a two-sided *P* ≤ 0.05 was considered statistically significant. Comparison of categorical variables was performed using the χ2 test. Independent predictive significance of risk factors identified by univariate analysis was computed by the Cox regression model. Spearman rank correlation analysis was used for nonparametric correlations. The progression-free survival was estimated using the Kaplan-Meier method, and comparison of survival rates among groups was conducted using the log-rank test. Data are expressed as mean ± standard deviation or as a percentage.

## SUPPLEMENTARY TABLES


