# Supplementary material for: pERK/pAkt phenotyping in circulating tumor cells as a biomarker for sorafenib efficacy in patients with advanced hepatocellular carcinoma
Source: Oncotarget. 2015 Oct 26;7(3):2646–59. doi: 10.18632/oncotarget.6104 (PMC4823061; doi:10.18632/oncotarget.6104)
Supplement: Supplementary file 1 [file oncotarget-07-2646-s001.pdf]

SUPPLEMENTARY TABLES

Supplementary Table S1: pERK/pAkt phenotyping in tumor tissues and circulating tumor cells (CTCs) from patients with hepatocellular carcinoma

Phenotype	Tumor tissues		CTCs	
	n	%	*n*	%
Total	32	100	101	100
$pERK^{+}/pAkt^{+}$	4	12.5	16	15.9
$pERK^{+}/pAkt^{-}$	3	9.4	9	8.9
$pERK^{-}/pAkt^{+}$	23	71.9	65	64.4
$pERK^{-}/pAkt^{-}$	2	6.3	11	10.9
$pERK^{+}$	7	21.9	25	24.8
$pAkt^{+}$	27	84.4	81	80.2

Supplementary Table S2: CTCs counts pre- and post-treatment with sorafenib in each subset of HCC patients.

Patient classification	CTC counts		Decline (%)
	Pre-treatment	Post-treatment	
$pERK^{+}/pAkt^{+}$ (*n* = 11)	64 ± 34	39 ± 21	38.3 ± 19.1
$pERK^{+}/pAkt^{-}$ (*n* = 7)	52 ± 6	21 ± 11	60.6 ± 17.9
$pERK^{-}/pAkt^{+}$ (*n* = 33)	58 ± 14	48 ± 14	18.3 ± 6.3
$pERK^{-}/pAkt^{-}$ (*n* = 8)	40 ± 16	34 ± 15	17.1 ± 7.3

Supplementary Table S3: CTC subtypes and progression-free survivals (PFS) in 15 patients with pERK$^+$/pAkt$^-$ CTCs

Patient No.	CTCs (*n*)					(pERK$^+$/pAkt$^-$) CTCs/total	PFS (mo)
	pERK$^+$/pAkt$^+$	pERK$^+$/pAkt$^-$	pERK$^-$/pAkt$^+$	pERK$^-$/pAkt$^-$	Total		
1	27	3	8	0	38	0.08	1.1
2	12	8	23	0	43	0.19	1.5
3	0	15	0	38	53	0.28	2.8
4	58	52	19	8	137	0.38	1.7
5	69	45	0	0	114	0.39	2.2
6	0	32	23	14	69	0.46	6.1
7	0	27	0	24	51	0.53	7.6
8	20	31	0	5	56	0.55	6.5
9	0	37	10	17	64	0.58	8.4
10	0	37	0	24	61	0.61	6.9
11	0	43	0	13	56	0.77	9.9
12	0	32	2	0	34	0.94	10.7
13	0	43	0	0	43	1.00	12.4
14	0	48	0	0	48	1.00	14.7
15	0	50	0	0	50	1.00	13.1

Supplementary Table S4: Clinical characteristics of 109 patients with hepatocellular carcinoma

Characteristic	*n*	%
Sex		
Male	84	77.1
Female	25	22.9
Age, y	57.5 ± 13.1	
> 50	73	67.0
≤ 50	36	33.0
Etiology		
HBV only	84	77.1
HCV only	4	3.7
HBV & HCV	5	4.6
Non-HBV, Non-HCV	16	14.7
Maximum tumor size, cm		
> 3	66	60.6
≤ 3	43	39.4
Number of tumors		
Single	10	9.2
Multiple	99	90.8
AFP level, ng/mL		
< 400	70	64.2
≥ 400	39	35.8
ECOG PS		
0	34	31.2
1	42	38.5
2	33	30.3
Child-Pugh class		
A	77	70.6
B	32	29.4
Portal vein thrombus		
Positive	78	71.6
Negative	31	28.4
TNM staging		
III	84	77.1
IV	25	22.9
BCLC stage		
B (intermediate)	31	28.4
C (advanced)	78	71.6

(*Continued*)

**Characteristic**	***n***	**%**
Previous therapy		
Surgical resection	32	29.4
TACE	21	19.3
Radiofrequency ablation	6	5.5
None	50	45.8

Abbreviations: AFP, alpha-fetoprotein; HBV, hepatitis B virus; HCV, hepatitis C virus; PS, performance status; TACE, transcatheter arterial chemoembolization; TNM, tumor-node-metastasis stage.
